# Inversion of Supramolecular Chirality by Sonication-Induced Organogelation

**DOI:** 10.1038/srep16365

**Published:** 2015-11-10

**Authors:** Sibaprasad Maity, Priyadip Das, Meital Reches

**Affiliations:** 1 Institute of Chemistry, The Hebrew University of Jerusalem, 91904, Jerusalem, Israel; 2 The Center for Nanoscience and Nanotechnology, The Hebrew University of Jerusalem, 91904, Jerusalem, Israel

## Abstract

Natural helical structures have inspired the formation of well-ordered peptide-based chiral nanostructures *in vitro*. These structures have drawn much attention owing to their diverse applications in the area of asymmetric catalysts, chiral photonic materials, and nanoplasmonics. The self-assembly of two enantiomeric fluorinated aromatic dipeptides into ordered chiral fibrillar nanostructures upon sonication is described. These fibrils form organogels. Our results clearly indicate that fluorine-fluorine interactions play an important role in self-assembly. Circular dichroism analysis revealed that both peptides (peptides **1** and **2)**, containing two fluorines, depicted opposite cotton effects in their monomeric form compared with their aggregated form. This shows that supramolecular chirality inversion took place during the stimuli-responsive self-aggregation process. Conversely, peptide **3**, containing one fluorine, did not exhibit chirality inversion in sonication-induced organogelation. Therefore, our results clearly indicate that fluorination plays an important role in the organogelation process of these aromatic dipeptides. Our findings may have broad implications regarding the design of chiral nanostructures for possible applications such as chiroptical switches, asymmetric catalysis, and chiral recognitions.

Helicity creates chiral motifs in numerous biomolecular structures. Two common helical structures are (1) the α-helix, a secondary structure of proteins, and (2) the double helix formed by the two strands of DNA^1^,^2^. These helical structures can interact with each other to form helical supramolecular assemblies^3^. In this manner, by hierarchical self-assembly, a coiled coil structure is formed in proteins or in the supercoils in DNA plasmids^4^. The helicity of these biopolymers is very important for maintaining their essential functions, which include replication, recognition, and selective catalytic activity^5^,^6^,^7^,^8^. Therefore, these natural structures have inspired researchers to design molecular building blocks that will form, through intermolecular non-covalent interactions, supramolecular helical nanostructures having versatile applications in different fields^9^,^10^,^11^,^12^,^13^,^14^.

Chiral interactions are very attractive due to their applications in biological processes^15^, medicine^16^, medicine and chemistry^17^,^18^. Supramolecular chirality specifies the induction or inversion of chirality based on the supramolecular arrangement of monomers through non-covalent interactions such as hydrogen bonding, van der Waals interactions, π–π stacking, hydrophobic interactions, and others. Among the various self-assembled systems, supramolecular gelators have a great tendency to form chiral aggregates owing to their self-assembly propensity through various non-covalent interactions^19^,^20^. The supramolecular chirality, however, does not depend on whether the monomer is chiral or achiral^21^,^22^. Instead, it is regulated by how the molecules are arranged in the self-assembled aggregates. Although various types of gelators have been shown to possess supramolecular chirality^23^,^24^,^25^,^26^, to date, very few peptide-based systems have been reported. For example, opposite supramolecular chirality (left- and right-handed) from porphyrin-conjugated pentapeptides in two different solvent systems were investigated by Jiang *et al.*^27^. Hirst *et al.* nicely demonstrated the effect of stereochemistry on a two-component organogel from two stereoisomeric lysine-based dendritic peptides^28^. Das *et al.* reported the formation of helical nanofibrils from the self-assembly of lysine-based peptide amphiphile (PA)^29^. In addition, using vibrational circular dichroism (VCD), Nafie *et al.* investigated the supramolecular chirality of polyglutamine^30^. Recently, Conejero-Muriel *et al.* have demonstrated the effect of the supramolecular chirality of short peptide hydrogels on protein crystallogenesis^31^. Moreover, the effects of solvents and the participation of chiral guests on the supramolecular chirality of peptide-based organogelators were reported previously^32^,^33^,^34^,^35^. Here, we report, for the first time, the inversion of chirality by fluorinated peptide-based gelators that contain two fluorine atoms, upon sonication-induced organogelation.

We chose to study aromatic peptides that contain fluorine atoms as substitutions. Fluorine has been extensively explored during the last decade for biotechnology uses and medicinal chemistry applications owing to its unique propensity to form extensive hydrogen bonds, as well as its biocompatibility and resistance to protease activities^36^,^37^. The formation of hydrogels by fluorinated aromatic peptides was previously reported by different groups^38^,^39^,^40^. Recently, Wu *et al.* demonstrated the effect of fluorination on the hydrogelation of 4-fluorobenzyl-capped diphenylalanine^41^. Here, we report on the sonication-induced organogelation of fluorinated dipeptides. The peptides containing two fluorine substitutions exhibited chiral inversion during their supramolecular assembly, whereas their mono-fluorinated analogue did not exhibit this behavior. To the best of our knowledge, this is the first report of supramolecular chirality inversion induced by the gelation of aromatic substituted fluorinated peptides.

## Results

### Preparation and characterization of the organogels

**Figure 1:**
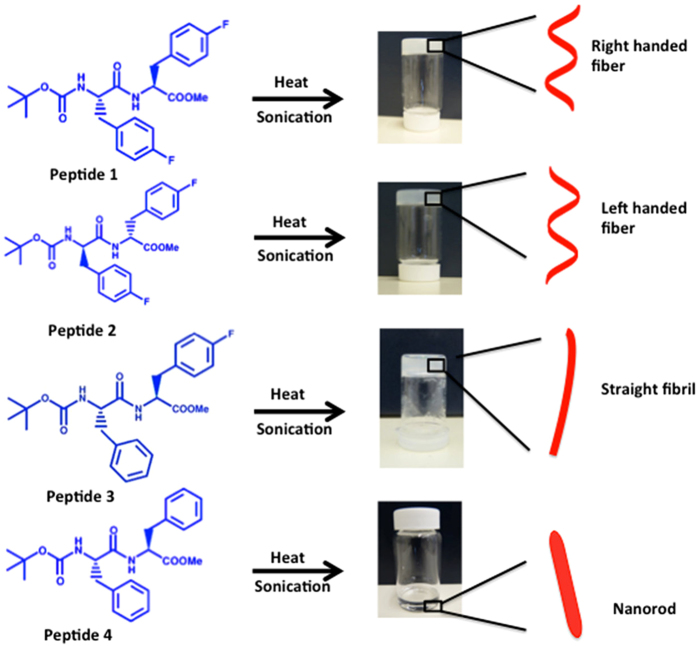
The chemical structure of peptides 1–4 and the schematic representation of the sonication-induced assemblies. The photographs show the organogels formed by peptides **1**, **2**, and **3** and the solution obtained after sonication of peptide **4**. Red helixes, lines, and rods represent helical fibers, straight fibers, and nanorods, respectively.

The gelator molecules (peptides **1**, **2**, and **3**, Fig. 1) were designed to ensure the maximum probability of self-assembly through hydrogen bonding, π-π stacking^60^,^61^, and hydrophobic interactions. The most electronegative atom, fluorine, was introduced into the peptide side chains to enhance hydrogen bonding and fluorine-fluorine interactions^38^,^39^. Moreover, the number of fluorine substitutions was varied (peptides **1** and **2** have two, peptide **3** has one, and peptide **4** has no fluorine substitutions) to determine the effect of fluorination on gelation. All dipeptides were synthesized by a solution phase method, as described in our previous report (Supplementary Information)^37^. The gelation propensity of peptides **1**–**4** was evaluated using various organic solvents (Supplementary Table S1). Upon sonication, peptides **1**, **2**, and **3** formed organogels in a hexane-ethyl acetate mixture (3:1 v/v) or toluene. The organogel in hexane-ethyl acetate (3:1 v/v) of peptide **1** and **3** formed an opaque gel, probably due to less ordered nanostructures, whereas the gel formed by peptide **2** was transparent (Fig. 1). Peptides **1** and **2**, having two fluorine substitutions, formed a gel instantly after 30 seconds of sonication, whereas peptide **3**, containing only one fluorine, formed a gel after 30 seconds of sonication, followed by keeping the system undisturbed for 2 hours at room temperature. Interestingly, their non-fluorinated analogue (peptide **4**) could not form a gel under identical experimental conditions. In other organic solvents such as hexane, cyclohexane, ethyl-acetate, benzene, nitrobenzene, tetrahydrofuran, and others the peptides were either soluble or precipitated out (Supplementary Table S1). The gelation of all gelators (peptides **1**, **2**, and **3**) was reversible upon heating and sonication. The gelation properties were examined by the tube inversion method^42^. The sol-gel transition temperatures (T_gel_) of the gels were 45 °C–46 °C and 48 °C–50 °C for peptide **1** (2.0 w%) and for peptide **2** (2.0 w%) in toluene, respectively. The observed T_gel_ temperature for the gel formed by peptide **3** (in toluene) was 41 °C–43 °C, which is comparatively lower than the T_gel_ values observed for peptides **1** and **2**. These results indicate that fluorination of aromatic peptides promotes the organogelation processes; however, fluorine substitution on both aromatic residues is more efficient. This is because the gelation in this case was faster and the T_gel_ was higher. A similar type of influence of halogenation on hydrogelation was reported recently by Bertolani *et al.*^43^.

**Figure 2:**
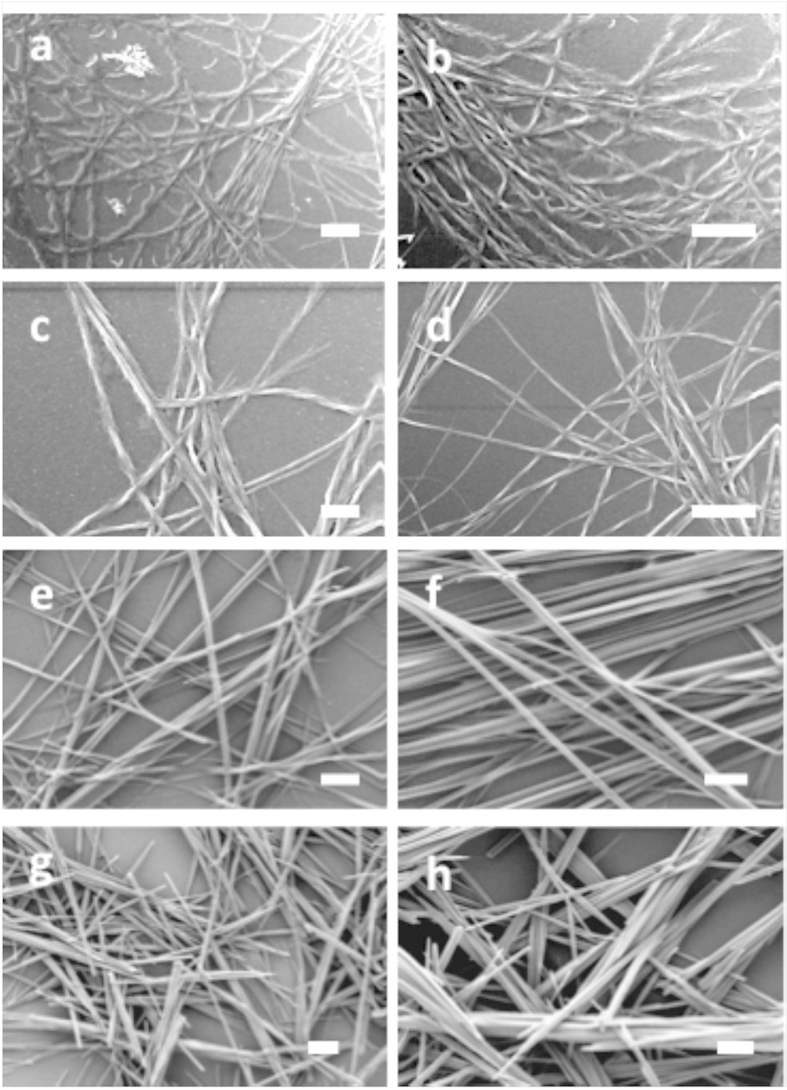
High-resolution scanning electron microscopy (HR-SEM) analysis of the peptide assemblies. (**a**,**b**) xerogel formed by peptide **1**, (**c**,**d**) xerogel formed by peptide **2**, (**e**,**f**) xerogels formed by peptide **3**; and (**g**,**h**) self-assembled aggregates formed by peptide **4**. For (**a**,**c**,**e**,**g**) the solvent was hexane-ethyl acetate mixture (3:1 v/v) and for (**b**,**d**,**f**,**h**) the solvent was toluene. The scale bars are 2 μm.

To obtain information on the microscopic structure of these organogels, we utilized high-resolution scanning electron microscopy (HR-SEM) and transmission electron microscopy (TEM). Figure 2a,b depict HR-SEM images of xerogels formed by peptide **1** in a hexane-ethyl acetate mixture (3:1 v/v) and toluene, respectively. These images clearly show the assembly of helical fibrils with a diameter of 100–200 nm, which form a fibrillar network. Similar types of elongated helical nanofibers (diameter range 120–200 nm) were also formed by peptide **2** using the same solvents (Fig. 2c,d). In contrast, peptide **3** formed non-helical fibrillar nanostructures (straight fibrils) (Fig. 2e,f), whereas peptide **4** formed non-helical needle-shaped nanostructures (nanorods) in these solvents under identical experimental conditions (Fig. 2g,h).

**Figure 3:**
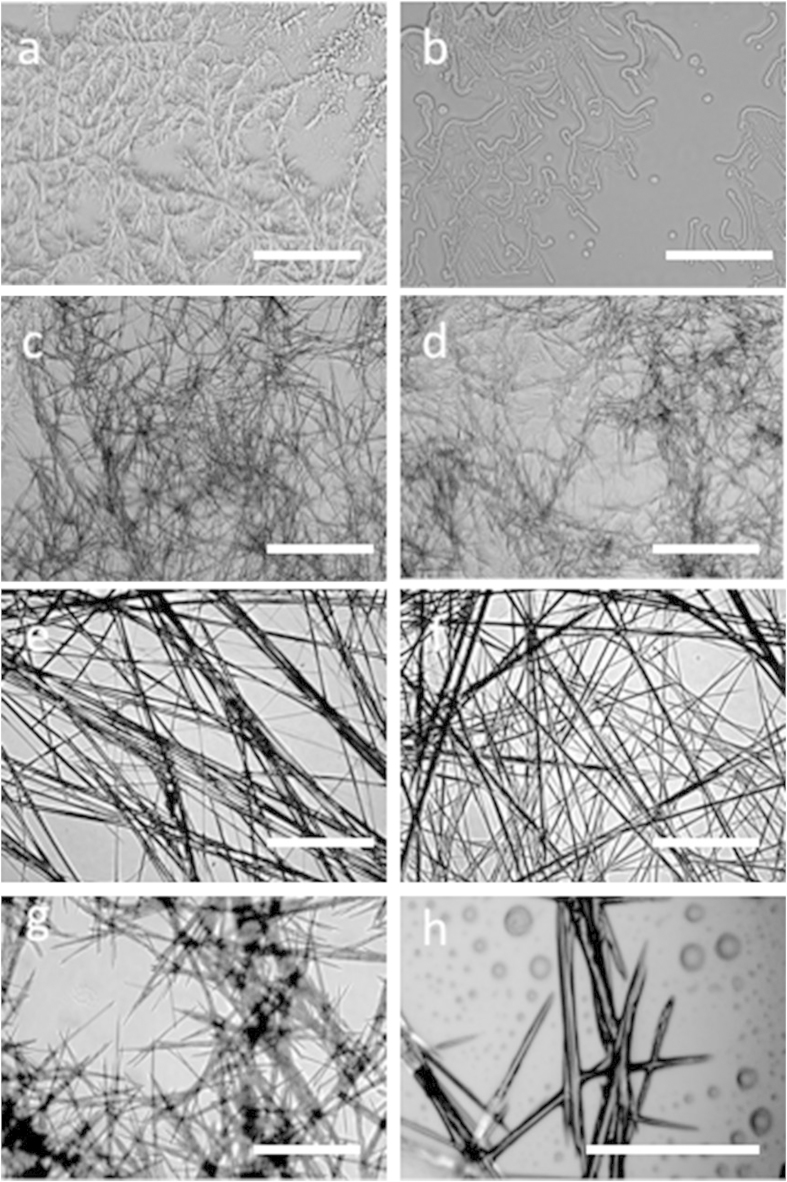
Optical microscopy images of the assemblies formed by sonication. Optical microscopy images of xerogels formed by (**a**) Peptide **1** in hexane-ethyl acetate (3:1 v/v) and (**b**) Peptide **1** in toluene; (**c**) Peptide **2** in hexane-ethyl acetate (3:1 v/v), and (**d**) Peptide **2** in toluene; (**e**) Peptide **3** in hexane-ethyl acetate (3:1 v/v), and (**f**) Peptide **3** in toluene; (**g**,**h**) representative images of sonication-induced self-assembled aggregates formed by peptide **4** in hexane-ethyl acetate (3:1 v/v) and toluene, respectively. Scale bars represent 20 μm.

TEM analysis also provided evidence for the formation of a fibrillar network for peptides **1** and **2** (Supplementary Figure S1). Figures S1a and S1b indicate that the xerogel formed by peptide **1** in hexane-ethyl acetate mixture (3:1 v/v) and toluene has thicker fibers (diameter range 300–400 nm), which were formed by the association of several thousand thinner fibrils (diameter ca. 10 nm). The xerogel formed by peptide **2** in both solvents exhibited fibrillar (diameter range 100–200 nm) networks (Supplementary Figure S1c,d). Optical microscopy analysis confirmed that the xerogels formed by peptides **1, 2**, and **3** originated from a fibrillar network (Fig. 3a–f). It also supported the formation of needle-like nanostructures by peptide **4** under the same experimental conditions (Fig. 3g,h).

### Mechanical properties

**Figure 4:**
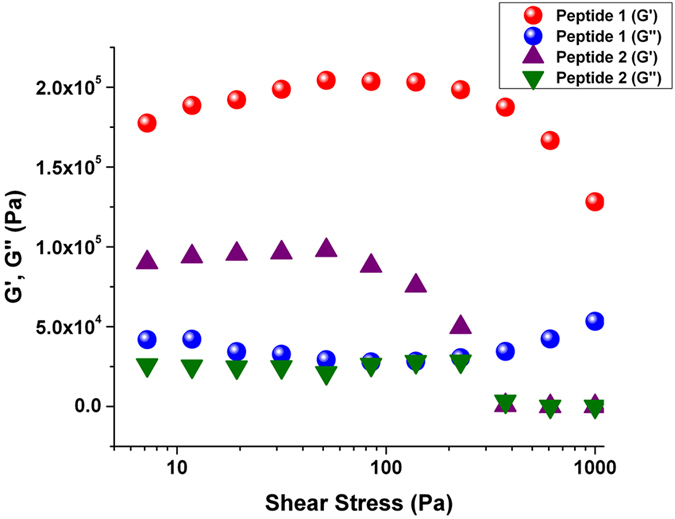
The mechanical properties of the organogels. Rheological study of the organogels formed by peptides **1** and **2** in toluene. The graph plots the dependence of the storage modulus (G′) and the loss modulus (G″) on the shear stress at a constant frequency of 1 Hz at 25°C.

The viscoelastic behavior of the organogels formed by peptides **1, 2**, and **3** were examined by rheological studies. The shear stress-sweep experiments were performed to evaluate the mechanical strength of the peptide-based organogels in toluene^44^. Figure 4 shows a dependence of the dynamic storage modulus (G′) and the loss modulus (G″) on the shear stress for both organogels formed by peptides **1** and **2** (3.0 wt%) at 25°C. We used toluene as a solvent because toluene is less volatile when compared with hexane-ethyl acetate. The larger values of G′, compared with G″, indicate that the peptide-based organogels are viscoelastic in nature^45^. Upon the gradual increase in the shear stress at a constant frequency of 1 Hz, the gel formed by peptide **1** exhibited a mechanical stability up to 139 Pa, whereas the gel formed by peptide **2** exhibited lower stability up to 53 Pa. The results also indicated that up to a given shear stress of 51 Pa, the storage modulus G′ for the gel formed by peptide **1** (204 kPa) is almost twofold greater than that formed by peptide **2** (97 kPa). Hence, the mechanical stability of the gel formed by peptide **1** is twofold greater than the gel formed by peptide **2**. The higher mechanical stability of peptide **1** (containing L-amino acids) compared with peptide **2** (containing D amino acids) could be because the incorporation of D-amino acids into the peptide backbone is likely to distort the molecular packing and thus, weaken the intermolecular interactions^46^. Furthermore, the mechanical stability of the gel formed by peptide **3** is much lower than that of the gels from peptides **1** and **2** (Supplementary Figure S2). This indicates that the number of fluorine substitutions on the peptide backbone plays an important role in maintaining the mechanical stability of the gel. More fluorination on the peptide backbone leads to greater gelation efficiency and mechanical stability.

### Spectroscopic analysis

**Figure 5:**
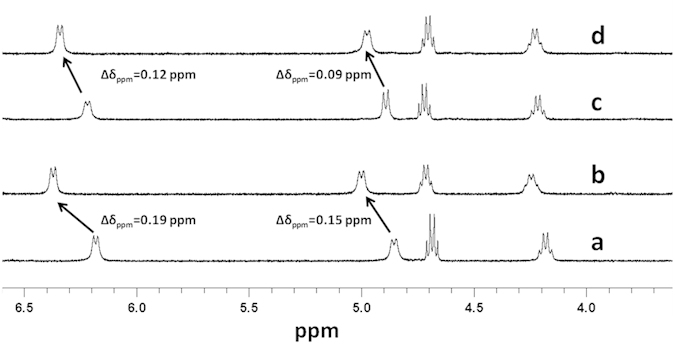
Changes in the proton NMR signals for the self-assembled gels. Downfield ^1^H NMR shift after sonication-induced gelation; ^1^H NMR spectra of peptide **1** (**a**) before sonication and (**b**) after sonication. Peptide **2** (**c**) before sonication and (**d**) after sonication in toluene d_8_ at room temperature. [Tetramethylsilane (TMS) as standard]

**Figure 6:**
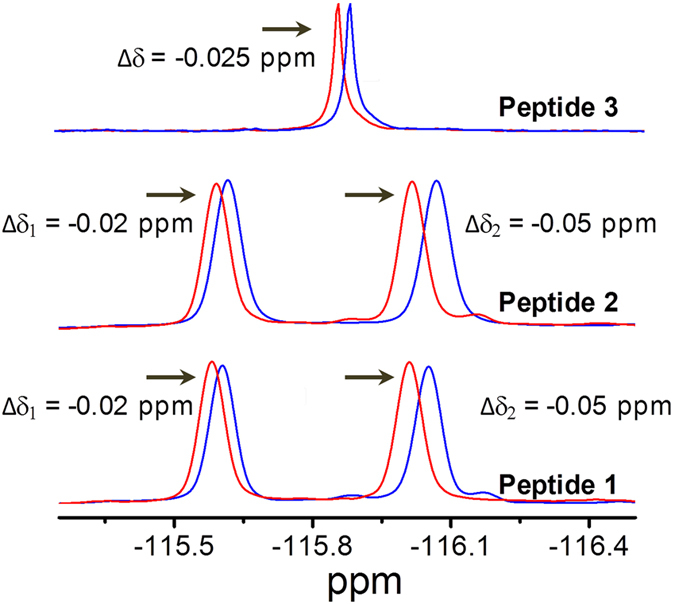
Changes in ^19^F NMR signals for the self-assembled gels. Up-field ^19^F NMR shift after the sonication-induced gelation of peptides **1**–**3**; red and blue lines represent ^19^F NMR before and after sonication, respectively. The data was recorded at room temperature in toluene d_8_. [Trichloro-fluoro methane (CFCl_3_) as standard]

To gain an insight into the mechanism underlying gel formation, we recorded ^1^H NMR spectra for peptides **1** and **2** before and after sonication in toluene-d_8_ (Fig. 5). For peptide **1**, the ^1^H NMR signals of NH Phe(1) and NH Phe(2) shifted downfield ∆δ = 0.15 ppm and ∆δ = 0.19 ppm, respectively, after sonication. The downfield shifting of amide protons indicates the participation of amide NH in intermolecular hydrogen bond formation. In the spectrum of peptide **2**, the downfield shifts of the ^1^H NMR signals of NH Phe(1) and NH Phe(2) were ∆δ = 0.09 ppm and ∆δ = 0.12 ppm, respectively. A similar type of amide NH shifting was observed during the sonication-induced self-assembly of Aβ peptide^18^,^19^,^20^,^47^. Furthermore, we also recorded the ^19^F NMR spectra to better understand the gelation mechanism. Up-field shifting of ^19^F NMR signals were detected upon sonication for peptides **1, 2**, and **3** in toluene d_8_ (Fig. 6). The ^19^F NMR signals of two F atoms had an up-field shift of ∆δ_1_ = 0.02 ppm and ∆δ_2_ = 0.05 ppm for peptide **1**. The detected up-field shifting (∆δ) values of ^19^F NMR fluorine signals for peptide **2**, upon sonication, were 0.02 ppm and 0.05 ppm, respectively. A similar type of up-field shift of the ^19^F NMR signal for the gel formed by peptide **3** was also observed (∆δ = 0.025 ppm) in toluene d_8_. The up-field shift of ^19^F NMR signals can be explained by the fact that fluorine-rich individual peptide fibers aggregated to form thicker fibrils by a fluorine-fluorine interaction (fluorous effect) during the sonication-induced gelation process. Similar types of fluorine-fluorine hydrophobic interactions were reported by Tan *et al.*^48^. The inter-fibril interaction to form thicker fibrils was also supported by HR-SEM (Fig. 2a–d) and TEM (Supplementary Figure S1a–d) analysis of the xerogels.

The effect of gelation on the C-F stretching frequency has been studied by FT-IR spectroscopy. The infrared absorption band ranging from the 1000–1400 cm^−1^ region is usually considered as the C-F stretching frequency for fluorinated compounds^49^. We compared the FT-IR spectra of the peptides in their monomeric form to their aggregated form (xerogels). To obtain the monomeric form of the peptides, we dissolved the peptides in 1,1,1,3,3,3 hexafluoro 2-propanol (HFP). This solvent interferes with intermolecular interactions and eliminates pre-aggregation^50^. The peptide solution and the xerogels formed in hexane-ethyl acetate were then drop-casted on a KBr FT-IR window. In the monomeric form, the C-F stretching frequency for peptide **1** exhibited a peak at 1164 cm^−1^; however, in xerogels the peak resolved into two clear peaks at 1166 cm^−1^ and 1155 cm^−1^, indicating that the fluorine atoms participated in the fibrilization processes (Supplementary Figure S3a). Similarly to peptide **1**, the monomeric form of peptide **2** exhibited a C-F stretching frequency at 1165 cm^−1^ and its xerogels exhibited two peaks at 1167 cm^−1^ and 1155 cm^−1^ (Supplementary Figure S3b). Peptide **3** did not exhibit a significant change in C-F stretching frequency (Supplementary Figure S3c) between its monomeric and xerogels form (both had a single peak at 1167 cm^−1^), which could be due to the medium resolution of the FT-IR spectroscopic method.

Fourier transform infra-red spectroscopy (FT-IR) can also be a valuable tool for determining the secondary structures of proteins and peptides^51^. The deconvoluted FT-IR spectra in the range 1600–1700 cm^−1^ provided valuable information on the peptide secondary structures. The spectrum of the xerogel of peptide **1** exhibited peaks at 1662 cm^−1^ and 1678 cm^−1^, which suggests a β-turn conformation (Supplementary Figure S4)^52^. The spectra of the xerogels formed by Peptide **2** and **3** exhibited peaks which also suggest a β-turn structure (Supplementary Figure S4). The FT-IR spectrum of the sonication-induced aggregates of peptide **4** suggests a mixture of α-helix and an anti-parallel β-sheet structure^53^ (amide I peaks at 1617 cm^−1^, 1634 cm^−1^, 1656 cm^−1^, and 1686 cm^−1^). Although this spectrum suggests more ordered structures for peptide **4**, it overall indicates a mixture of structures which may lead to a less ordered state. In addition, it should be noted that this method has only a medium resolution.

### Chirality Assays

**Figure 7:**
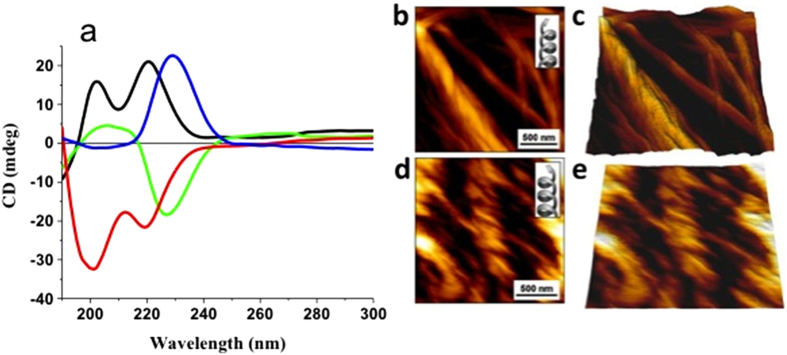
Supramolecular chirality inversion measurements. Solid-state CD spectra of (**a**) peptide **1** in HFP (black), peptide **1** xerogel (2 wt%) in a hexane-ethyl acetate mixture (3:1 v/v) (green), peptide **2** in HFP (red), and peptide **2** xerogel (2 wt%) in a hexane-ethyl acetate mixture (3:1 v/v) (blue). AFM micrographs of peptide **1** xerogel (**b**,**c**); peptide **2** xerogel (**d**,**e**) formed in a hexane ethyl acetate mixture (3:1 v/v). The Z-scale for the 3-dimensional AFM micrographs (**c,e**) is 125 nm.

We investigated the supramolecular chirality of the peptides by solid-state circular dichroism (CD) measurements to this end, CD spectra of the monomeric peptides were compared with their corresponding xerogel aggregates for all gelators. The CD spectrum of peptide **1** in its monomeric form had two positive peaks at 202 nm and 220 nm. This indicates a β-turn structural conformation (Fig. 7a)^54^,^55^. Surprisingly, the spectrum of the xerogel of peptide **1** from the hexane-ethyl acetate mixture (3:1 v/v) had a negative peak at 227 nm, indicating a supramolecular assembly in the gel state^56^. Similar types of CD spectra were obtained for quadruple helix, which were reported by Stupp *et al.*^57^. The CD spectra of peptide **2** showed a similar trend. The CD spectrum of the monomeric form of peptide **2** exhibited two negative peaks at 200 nm and 219 nm, and the spectrum of the xerogels had one positive signal at 229 nm. The cotton effect, which we observed for the self-assembled xerogels, compared with their monomeric form, clearly indicates that chirality inversion exists during the self-assembly of peptides **1** and **2**^58^. Thus, we suggest that chirality inversion occurs due to the supramolecular arrangement of the monomers during the gelation process. This type of chirality inversion during the gelation of fluorinated peptides is very uncommon. Inversion of supramolecular chirality was further visually tested by atomic force microscopy (AFM)^59^. Two-dimensional (2D, Fig. 7b) and three-dimensional (3D, Fig. 7c) images clearly indicate right-handed helical fibers for the xerogel formed by peptide **1 (**composed of L-amino acids). In contrast, left-handed helical fibers were clearly detected for the xerogel formed by peptide **2** (composed of D-amino acids) in the 2D (Fig. 7d) and 3D (Fig. 7e) AFM micrographs. Hence, the AFM analysis is in good agreement with the supramolecular chirality inversion detected by the CD measurements. Interestingly, peptide **3**, with one fluorine substitution, did not exhibit chirality inversion behavior under similar conditions. The CD spectra of peptide **3**, in its monomeric form, exhibited two strong positive peaks at 220 nm, corresponding to the n-π* transition, and at 197 nm, corresponding to the π-π* transition, whereas the corresponding xerogel obtained from hexane-ethyl acetate (3:1 v/v) exhibited one positive peak at 224 nm, indicating a supramolecular assembly in the gel state (Supplementary Figure S5).

## Discussion

**Figure 8:**
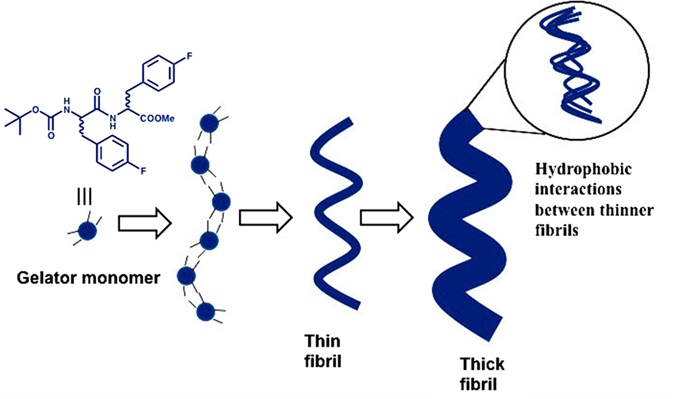
Schematic representation of the self-assembly process responsible for organogelation. The black line in the monomer model represents binding sites. The thinner fibrils associate with each other by fluorine-fluorine interactions, resulting in thicker fibrils.

We demonstrated the design and synthesis of fluorinated dipeptides, which form organogel upon sonication. The fluorine atoms were introduced intentionally in the peptide residues to enhance the interfibrillar interactions. The non-fluorinated analogue (peptide **4)** did not form a gel, whereas peptides **1, 2**, and **3** with fluorine substitutions formed well-defined gels upon heating and sonication. Peptides **1** and **2** (with two fluorine substitutions) exhibited more gelation efficiency than peptide **3** (with only one fluorine substitution). Our results clearly indicate that the fluorination of peptides improves their ability to form gels by enhancing the probability of hydrogen bonding and fluorine-fluorine interactions. CD and AFM topography analysis revealed that chirality inversion took place during the self-assembly of peptides **1** and **2** into gels. Moreover, the structural analysis (Figs 2, 3 and 7) and the NMR studies (Figs 5 and 6) shed light on the mechanism underlying the organogelation process for peptides **1** and **2**. The downfield shifting of the amide proton clearly indicated intermolecular hydrogen bond formation, whereas the up-field shifting of the ^19^F signals confirmed a fluorine-fluorine interaction during the organogelation process. HR-SEM and TEM micrographs clearly indicated that many thin fibers are associated with thicker fibrils in the xerogels. Based on these results, we proposed a mechanism underlying organogelation, which is pictorially shown in Fig. 8. According to our proposed model, the monomers initially self-assemble into helical thin fibrils through hydrogen bonding (as shown by ^1^H NMR analysis) and aromatic interactions, which then associate to create thicker fibrils via hydrophobic interactions (as shown by ^19^F NMR analysis). These interactions enabled the peptides to form organogels. We suggest that supramolecular association of the needle-shaped nanorods formed by peptide **4** did not take place due to the absence of fluorine; therefore, peptide **4** could not generate an organogel.

In conclusion, we demonstrated a stimuli-responsive organogel system formed by fluorinated dipeptides. Upon sonication, the fluorinated dipeptides formed organogels, whereas their non-fluorinated analogue did not. We have shown that fluorine-fluorine interactions play an important role in the gelation propensity of gelators. More interestingly, supramolecular chirality inversion took place during sonication-induced organogelation, which is very rare in gel-based systems. These findings provide an important insight into the design of chiral gels.

## Methods

### Materials

All chemicals and solvents are commercially available and were used as supplied unless otherwise stated. All amino acids (L-phenylalanine, L & D-4 fluoro phenylalanine) were purchased from Chem Impex, Inc. (Wood Dale, IL, USA). 1-Hydroxybenzotriazole (HOBt) and dicyclohexylcarbodiimide (DCC) were purchased from Alfa-Aesar (Ward Hill, MA, USA). All NMR solvents were purchased from Sigma-Aldrich (St. Louis, MO, USA).

### Peptide synthesis

The peptides were synthesized by conventional solution-phase methodology. The Boc group was used for N-terminal protection, and the C-terminus was protected as a methyl ester. Couplings were mediated by DCC/HOBt. All the intermediates were characterized by 500 MHz and 400 MHz ^1^H NMR, and MALDI-Tof mass spectrometry. The final compounds were fully characterized by ^1^H NMR, ^13^C NMR, and ^19^F NMR (for fluorine-containing peptides) spectroscopy, MALDI-Tof mass spectrometry, or LC-MS.

### Self-assembly

The peptides were dissolved in either hexane-ethyl acetate or in toluene at their minimum gelation concentrations, and then heated and sonicated for 30 sec. A stable organogel was obtained instantly from peptides **1** and **2**. Peptide **3**, containing only one fluorine substitution, formed a gel after 30 sec of sonication and after 2h of curing time at room temperature. Their non-fluorinated analogue (peptide **4**) did not form any gel under similar experimental conditions.

### High-Resolution Scanning Electron Microscopy (HR-SEM)

The gel samples were placed on clean glass cover slips and dried under vacuum. The samples were then coated with gold using a Polaron SC7640 Sputter Coater and imaged using a high-resolution scanning electron microscope, Sirion, operating at 5 kV.

### Transmission Electron Microscopy (TEM)

The peptides were suspended in either toluene (20 mg/mL) or hexane-ethyl acetate (10 mg/mL), heated, and sonicated for 30 sec. Then, a 10 μl drop was placed on a carbon-coated TEM grid (200 mesh, Electron Microscopy Science, PA, USA) and dried under vacuum. The images were acquired using a TEM instrument (Tecnai T12 G2 Spirit (cryoTEM)) operating at 120 kV.

### Nuclear Magnetic Resonance (NMR)

The synthetic peptides and their intermediates were dissolved in either CDCl_3_ or DMSO. The shifting of the amide protons and fluorine signals during the self-assembly process was also determined by NMR spectroscopy. The proton NMR was acquired by dissolving 10 mg of the fluorinated dipeptides into 0.6 mL of toluene d_8_ in an NMR tube before and after sonication. ^19^F NMR was also acquired similarly before and after sonication. All the NMR data were acquired in either a Bruker Avance 400 MHz or 500 MHz spectrometer.

### Fourier Transform Infrared spectroscopy (FT-IR)

A 20 μL peptide solution in HFP (10 mg/mL) was drop-casted on KBr windows and dried under vacuum. To measure the xerogels, the organogels formed by the peptides were dropped onto KBr windows and dried under vacuum. FT-IR spectra were recorded using a Nicolet 6700 FT-IR spectrometer (Thermo Fisher Scientific, Waltham, MA, USA). The measurements were taken at 4 cm^−1^ resolution and with an average of 3000 scans. The FT-IR spectra were de-convoluted using a Gaussian function of MagicPlot software.

### Atom Force Microscopy (AFM)

The gels were placed on clean glass coverslips and dried under vacuum. Topography images of the structures were taken using a JPK NanoWizard®3 (JPK Instruments, Berlin, Germany) working in AC mode. Si_3_N_4_ cantilever probes with a nominal spring constant of 3 Nm^−1^ and a resonance frequency of 75 kHz were used.

### Circular Dichroism (CD) spectroscopy

The CD spectra of the peptides were recorded using a JASCO J-810 Spectrophotometer (JASCO, Japan) and analyzed with the supplied Spectra-Manager software. Each of the peptides was dissolved in HFP to a concentration of 10 mg/mL. Then, a 20 μL drop of the HFP solution was placed on a quartz slide and dried under vacuum. To analyze the xerogel, 8 mg of the peptide was suspended in 500 μL of a hexane-ethyl acetate mixture (3:1 v/v), heated and sonicated for 30 seconds, drop-casted on a quartz glass, and finally dried in vacuum for 3 hours. The spectrum was then recorded.

### Rheology measurement

Rheological measurements were performed using a Rheoscope 1 rheometer (Thermo-Haake, Karlsruhe, Germany). A cone−plate sensor was used with a diameter of 35 mm, a cone angle of 1°, and a gap of 0.024 mm. The viscoelastic properties of organogels were determined with increasing stress at a constant frequency of 1Hz at 25 °C.

## Additional Information

**How to cite this article**: Maity, S. *et al.* Inversion of Supramolecular Chirality by Sonication-Induced Organogelation. *Sci. Rep.* **5**, 16365; doi: 10.1038/srep16365 (2015).

## Supplementary Material

Supplementary Information
