# Supplementary material for: Inversion of Supramolecular Chirality by Sonication-Induced Organogelation
Source: Sci Rep. 2015 Nov 10;5:16365. doi: 10.1038/srep16365 (PMC4639836; doi:10.1038/srep16365)
Supplement: Supplementary Information [file srep16365-s1.doc]

# Supplementary information

# Inversion of Supramolecular Chirality by Sonication-Induced Organogelation

Sibaprasad Maity^1,2^, Priyadip Das^1,2^, and Meital Reches^1,2^*

#

1. Institute of Chemistry, The Hebrew University of Jerusalem, 91904 Jerusalem, Israel.

2. The Center for Nanoscience and Nanotechnology, The Hebrew University of Jerusalem, 91904 Jerusalem, Israel.

*Corresponding Authors:

Meital Reches, E-mail: [meital.reches@mail.huji.ac.il](mailto:meital.reches@mail.huji.ac.il), Tel: +972-2-6584551, Fax: +972-2-6584501.

**Table S1**. Gelation assays in different organic solvents. The table indicates the appearance of the solution/gels, the minimum gelation concentrations (mgc), and the sol-gel transition temperature (T_gel_) for the gels.

| **Gelator** | **Solvent** | **Appearance** | **mgc (wt %)** | **T_gel_ (°C) at mgc** |
| --- | --- | --- | --- | --- |
| Peptide **1**  Peptide **2**  Peptide **3** | n-Hexane | Precipitation  Precipitation  Precipitation | –  –  – | –  –  – |
| Peptide **1**  Peptide **2**  Peptide **3** | Ethyl acetate | Soluble  Soluble  Soluble | –  –  – | –  –  – |
| Peptide **1**  Peptide **2**  Peptide **3** | n-Hexane : Ethyl acetate (3:1) | Gel  Gel  Gel | 1  1  2 | 44°C-46°C  47°C-48°C  40°C -42°C |
| Peptide **1**  Peptide **2**  Peptide **3** | Benzene | Soluble  Soluble  Soluble | –  – | –  –  – |
| Peptide **1**  Peptide **2**  Peptide **3** | Toluene | Gel  Gel  Gel | 2  2  2 | 45°C-46°C  48°C-50°C  41°C -43°C |
| Peptide **1**  Peptide **2**  Peptide **3** | Cyclohexane | Precipitation  Precipitation  Precipitation | –  –  – | –  –  – |
| Peptide **1**  Peptide **2**  Peptide **3** | Nitrobenzene | Precipitation  Precipitation  Precipitation | –  –  – | –  –  – |
| Peptide **1**  Peptide **2**  Peptide **3** | Tetrahydrofuran | Soluble  Soluble  Soluble | –  –  – | –  –  – |
| Peptide **1**  Peptide **2**  Peptide **3** | Acetonitrile | Soluble  Soluble  Soluble | –  –  – | –  –  – |
| Peptide **1**  Peptide **2**  Peptide **3** | Dimethylsulphoxide | Soluble  Soluble  Soluble | –  –  – | –  –  – |

**
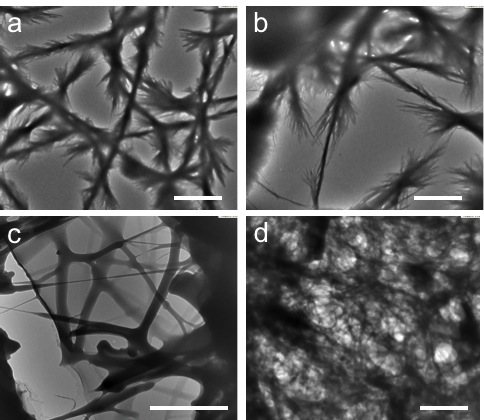
**

***Figure S1***. TEM images of the xerogels formed by (a) peptide **1** in hexane- ethyl acetate (3:1 v/v); (b) peptide **1** in toluene; (c) peptide **2** in hexane-ethyl acetate (3:1 v/v); and (d) peptide **2** in toluene; scale bars represent 2µm.


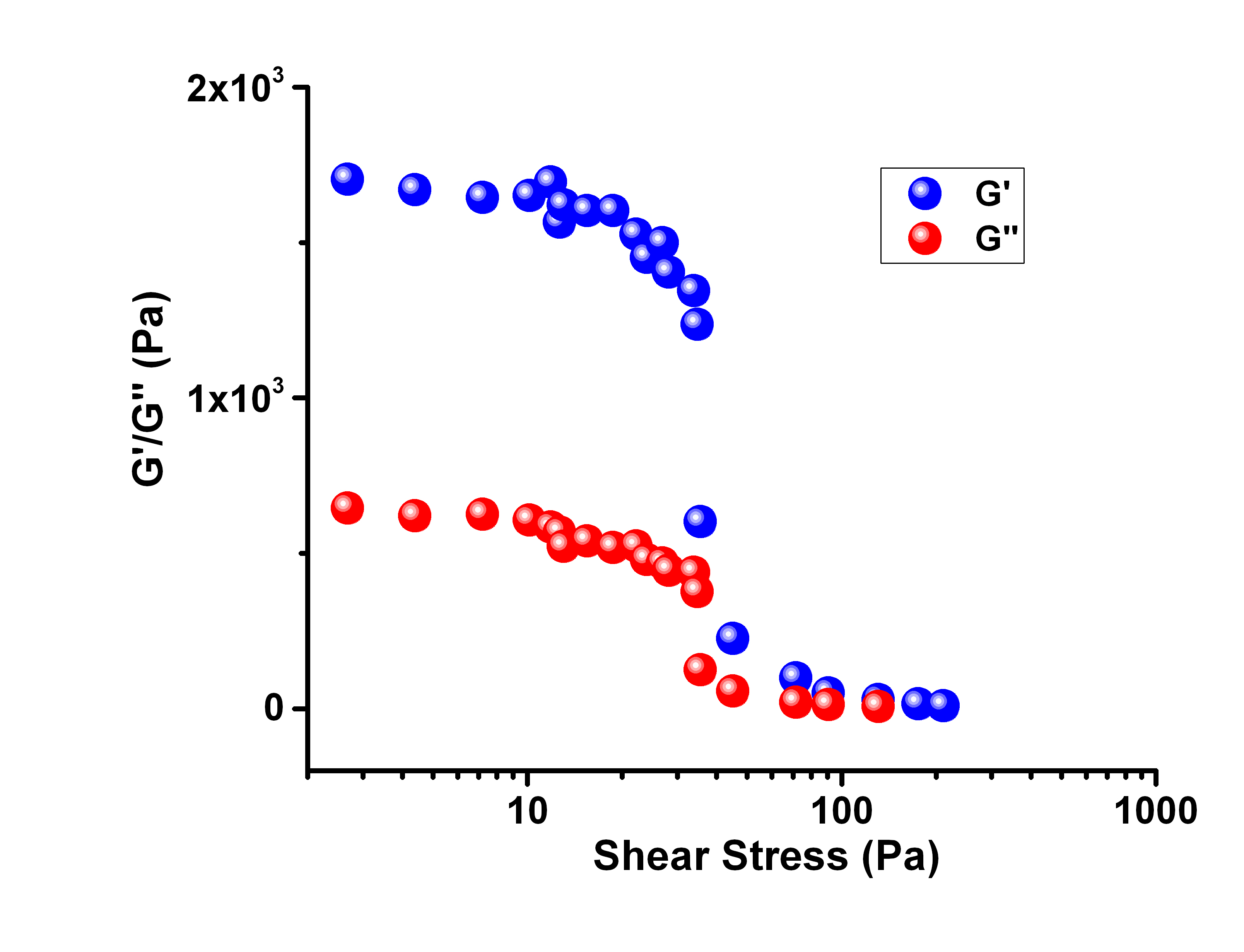


***Figure S2***. Rheological properties of the organogel formed by peptide **3** in toluene at room temperature.

***Figure S3***. FTIR spectra in the range 1000-1400 cm^-1^ for (a) peptide **1**, (b) peptide **2**, and (c) peptide **3**; the black line denotes the monomer form, and the red line denotes the xerogel form from hexane-ethyl acetate (3:1 v/v).

***Figure S4***. De-convoluted FT-IR spectra of a dried sample of the peptide xerogels from hexane-ethyl acetate (3:1 v/v) (a) peptide **1**; (b) peptide **2**; (c) peptide **3**; (d) peptide **4**. Deconvolution was done by Gaussian function. The solid lines with dots represent the original FT-IR spectra and the solid lines represent de-convoluted spectra.

***Figure S5***. Solid-state CD spectra for peptide **3**; the red and blue lines indicate the CD spectra of the monomers in HFP, and the xerogel formed in hexane-ethyl acetate (3:1 v/v), respectively.

**Details of peptide synthesis**

All the peptides were synthesized by conventional solution-phase methodology according to scheme S1.


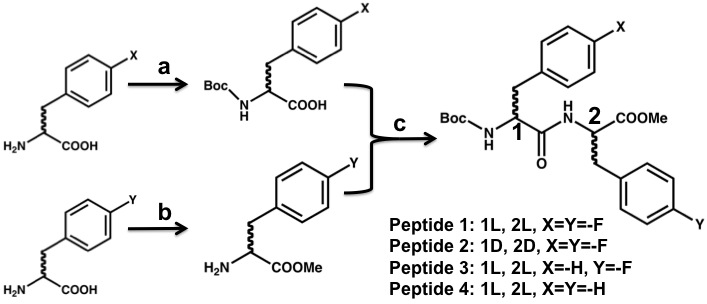


***Scheme S1***. Schematic route of the synthesis of peptide **1**-**4**; Reagents and conditions: (a) Boc anhydride, 1N NaOH, Dioxane-water, 0°C, overnight; (b) Thionyl chloride, methanol, 0°C, overnight, basify with 1N sodium bicarbonate solution and extract with ethyl acetate; (c) DCC, HOBt, Dry DCM, 0°C, 48h.

**A. Synthesis of Peptide 1**

**A1. Boc-L-(4F)Phe-COOH**: A solution of L-(4F)Phe-COOH 985 mg (5 mmol) in a mixture of dioxane (10 mL), water (5 mL), and 1 M NaOH (5 mL) was added, stirred, and cooled in an ice-water bath. This was followed by the addition of Ditert-butylpyrocarbonate (Boc anhydride) 1.2 g (5.5 mmol) and stirring at room temperature overnight. Then the solution was concentrated under vacuum to about 10 mL and cooled in an ice water bath. The aqueous phase was then covered with a layer of ethyl acetate (about 20 mL) and a dilute solution of KHSO_4_ was added slowly (in a drop wise manner) to acidify (pH 2–3). The aqueous phase was then extracted with ethyl acetate and this operation was repeated three times. The ethyl acetate extracts were then collected and dried over anhydrous Na_2_SO_4_ and evaporated in a vacuum. The pure material was obtained as a waxy solid.

Yield: 1.12 g (3.95 mmol, 79.0%)

^1^H NMR (DMSO-*d*_6_, 400 MHz, δ_ppm_): 12.60 [s, 1H COOH], 7.29-7.25 & 7.11-7.07 [m, 4H, Aromatic protons], 4.10-3.00 [m, 1H, CαH 4F Phe], 3.03-2.77 [m, 2H, CβH 4F Phe], 1.33 [s, 9H, Boc].

MALDI-TOF (matrix:α-cyano-4-hydroxy cinnamic acid (CHCA)):m/z= [M+H]^+^ 284.12 (calculated), 284.29 (observed), [M+Na]^+^ 306.11 (calculated), 306.25 (observed).

**A2. Boc-L-(4F)Phe(1)-L-(4F)Phe(2)-COOMe (1)**: 1.0 g (3.53 mmol) of Boc-L-(4F)Phe-OH was dissolved in 25 mL dry DCM in a round bottom flask fitted with a guard tube, cooled in an ice-water bath. Then 1.39 g (7.1 mmol) of NH_2_-L-(4F)Phe-OMe was added to the reaction mixture, which was isolated from the corresponding methyl ester hydrochloride by neutralization with a dilute solution of NaHCO_3_, and subsequently extracted with ethyl acetate followed by solvent evaporation. Then 1.1g (5.3 mmol) dicyclohexylcarbodiimide (DCC) and 717.0 mg (5.3 mmol) of HOBt were added as coupling reagents. The reaction mixture was allowed to come to room temperature and was stirred for 48 h. DCM was evaporated and the residue was re-dissolved in ethyl acetate (60 mL) and dicyclohexyl urea (DCU) was ﬁltered off. The organic layer was washed with 2 M HCl (2 × 30 mL), then brine (2 × 30 mL), then 1 M sodium carbonate (2 × 30 mL), and finally with brine (2 × 30 mL). All the collected ethyl acetate extracts were dried over anhydrous sodium sulfate and evaporated under vacuum The product was then puriﬁed by using a silica gel (100–200 mesh) column using n hexane–ethyl acetate (3 : 1) as eluent.

Yield: 1.23 g (2.66 mmol, 75.4%)

^1^H NMR (CDCl_3_, 400 MHz, δ_ppm_): 7.16-7.12 & 6.99-6.90 [m, 8H, Aromatic protons], 6.27-6.25 [d, *J*=8.0 Hz, 1H, NH 4F Phe(2)], 4.93 [b, 1H, NH 4F Phe(1)], 4.77-4.72 [m, 1H, CαH 4F Phe(2)], 4.28-4.27 [m, 1H, CαH 4F Phe(1)], 3.67 [s, 3H, OMe], 3.08-2.98 [m, 4H, CβH 4F Phe(1) and 4F Phe(2)], 1.41 [s, 9H, Boc]. ^13^C NMR (CDCl_3_, 125 MHz, δ_ppm_): 171.25, 170.74, 163.0, 162.91, 161.04, 160.96, 132.19, 131.37, 131.34, 130.88, 130.82, 130.79, 130.72, 115.52, 115.35, 55.78, 53.28, 52.37, 31.16, 28.25. ^19^F NMR (DMSO-d6, 470 MHz, δppm): -115.57, -116.00. FT-IR (cm^-1^) 3331, 1740, 1662, 1510, 1442, 1222, 1189.

MALDI-TOF (matrix:α-cyano-4-hydroxy cinnamic acid (CHCA)) m/z= [M+Na]^+^ 485.18 (calculated), 486.09 (observed), [M+K]^+^ 501.16 (calculated), 502.02 (observed).

**B. Synthesis of peptide 2**

**B1. Boc-D-(4F)Phe-COOH:** The compound was synthesized following a procedure similar to that used with Boc-L-(4F)-Phe-COOH**.**

^1^H NMR (DMSO-*d*_6_, 400 MHz, δ_ppm_): 12.59 [s, 1H COOH], 7.29-7.26 & 7.12-7.08 [m, 4H, Aromatic protons], 4.10-3.57 [m, 1H, CαH 4F Phe], 3.03-2.77 [m, 2H, CβH 4F Phe], 1.32 [s, 9H, Boc].

MALDI-TOF (matrix:α-cyano-4-hydroxy cinnamic acid (CHCA)):m/z= [M+H]^+^ 284.12 (calculated), 284.36 (observed), [M+Na]^+^ 306.11 (calculated), 306.28 (observed).

**B2. Boc-D-(4F)Phe(1)-D-(4F)Phe(2)-COOMe 2**: The peptide **2** was synthesized using the same procedure as with peptide **1**.

^1^H NMR (CDCl_3_, 400 MHz, δ_ppm_): 7.18-7.15 & 7.01-6.92 [m, 8H, Aromatic protons], 6.25-6.23 [d, *J*= 8.0 Hz, 1H, NH 4F Phe(2)], 4.93 [b, 1H, NH 4F Phe(2)], 4.77-4.76 [m, 1H, CαH 4F Phe(1)], 4.3-4.28 [m, 1H, CαH 4F Phe(2)], 3.7 [s, 3H, OMe], 3.1-3.0 [m, 4H, CβH 4F Phe(1) and 4F Phe(2)], 1.4 [s, 9H, Boc]. ^13^C NMR (CDCl_3_, 125 MHz, δ_ppm_): 17.24, 170.74, 163.0, 161.05, 160.96, 132.19, 131.37, 131.34, 130.88, 130.82, 130.79, 130.72, 115.51, 115.35, 53.28, 53.27, 52.37, 37.16, 33.97, 28.25, 25.64, 24.97. ^19^F NMR (DMSO-d6, 470 MHz, δ_ppm_): -115.57, -116.00. FT-IR (cm^-1^) 3332, 1740, 1662, 1510, 1445, 1220, 1158.

MALDI-TOF (matrix:α-cyano-4-hydroxy cinnamic acid (CHCA)):m/z= [M+Na]^+^ 485.18 (calculated), 486.13 (observed), [M+K]^+^ 501.16 (calculated), 502.03 (observed).

**C. Synthesis of peptide 3**

**C1. Boc-L-Phe-COOH:** The compound was synthesized using the same procedure as with Boc-L-(4F)-Phe-COOH.

^1^H NMR (DMSO-*d*_6_, 400 MHz, δ_ppm_): 12.654 [ b, 1H, COOH], 7.29-7.18 [m, 5H, aromatic protons], 7.11-7.09 [d, 1H, NH], 4.11-4.02 [m, 1H, CαH], 3.03-2.81 [dd, 2H, CβH], 1.25 [s, 9H, Boc].

MALDI-TOF (matrix:α-cyano-4-hydroxy cinnamic acid (CHCA)):m/z = [M+Na]^+^ 288.12 (calculated), 290.23 (observed).

**C2. Boc-L-Phe-L-(4F)Phe-COOMe 3**: The peptide **3** was synthesized using the same procedure as with peptide **1**.

^1^H NMR (CDCl_3_, 500 MHz, δ_ppm_): 7.30-7.18 & 6.96-6.89 [m, 9H, Aromatic protons], 6.32 [d, *J*=8.0 Hz, 1H, NH 4F Phe(2)], 4.96 [b, 1H, NH 4F Phe(1)], 4.77-4.74 [m, 1H, CαH 4F Phe(2)], 4.34-4.33 [m, 1H, CαH Phe(1)], 3.67 [s, 3H, OMe], 3.07-2.98 [m, 4H, CβH Phe(1) and 4F Phe(2)], 1.41 [s, 9H, Boc]. ^13^C NMR (CDCl_3_, 125 MHz, δ_ppm_): 171.21, 170.83, 162.98, 161.03, 155.33, 136.46, 131.40, 131.37, 130.80, 130.73, 129.36, 128.71, 127.03 115.49, 115.32, 80.33, 55.76, 53.30, 52.34, 38.17, 37.17 28.25. ^19^F NMR (DMSO-d6, 470 MHz, δppm): -115.85. FT-IR (cm^-1^) 3335, 1742, 1666, 1518, 1448, 1372, 1294, 1249, 1218 1170. LC-MS data m/z= [M+Na]^+^ 467.48 (calculated), 467.25 (observed).

**D. Synthesis of peptide 4**

**D1. Boc-L-Phe(1)-L-Phe(2)-COOMe 4:** The peptide **4** was synthesized using the same procedure as with peptide **1**.

^1^H NMR (CDCl_3_, 500 MHz, δ_ppm_): 7.31-7.21 & 7.03-7.01 [m, 10H, aromatic protons], 6.38-6.37 [d, *J*= 6.6 Hz, 1H, NH Phe(2)] 5.03 [b, 1H, NH Phe(1)], 4.82-4.81 [m, 1H, CαH Phe (1)], 4.38 [m, 1H, CαH Phe (2)], 3.69 [s, 3H, OMe], 3.09-3.06 [m, 4H, CβH Phe(1) & Phe(2)], 1.43 [s, 9H, Boc]. ^13^C NMR (CDCl_3_, 125 MHz, δ_ppm_): 171.29, 170.71, 136.46, 135.58, 129.29, 129.15, 128.57, 127.03, 80.07, 55.59, 53.20, 52.17, 37.89, 28.16. FT-IR (cm^-1^) 3228, 1742, 1657, 1697, 1662, 1523, 1445, 1341, 1250, 1168. MALDI-TOF [matrix:α-cyano-4-hydroxy cinnamic acid (CHCA)]: m/z = [M+Na]^+^ 449.20 (calculated), 450.44 (observed), [M+K]^+^ 465.18 (calculated), 466.41 (observed).

***Figure S6***. ^1^H NMR (DMSO-*d*_6_, 400 MHz, δ_ppm_) Boc-L-(4F)Phe-COOH.

***Figure S7***. ^1^H NMR (CDCl_3_, 400 MHz, δ_ppm_) of Boc-L-(4F)Phe(1)-L-(4F)Phe(2)-COOMe (**1)**.

***Figure S8***. ^13^C NMR (CDCl_3_, 125 MHz, δ_ppm_) of Boc-L-(4F)Phe(1)-L-(4F)Phe(2)-COOMe (**1)**.

***Figure S9***. ^19^F NMR (Toluene d_6_, 470 MHz, δ_ppm_) for Boc-L-(4F)Phe(1)-L-(4F)Phe(2)-COOMe (**1)**.

***Figure S10***. MALDI-TOF mass spectroscopy of Boc-L-(4F)Phe(1)-L-(4F)Phe(2)-COOMe (**1)**.

***Figure S11***. ^1^H NMR (DMSO-*d*_6_, 400 MHz, δ_ppm_) Boc-D-(4F)Phe-COOH.

***Figure S12*.** ^1^H NMR (CDCl_3_, 400 MHz, δ_ppm_) Boc-D-(4F)Phe(1)-D-(4F)Phe(2)-COOMe (**2)**.

***Figure S13***. ^13^C NMR (CDCl_3_, 125 MHz, δ_ppm_) of Boc-D-(4F)Phe(1)-D-(4F)Phe(2)-COOMe (**2)**.

***Figure S14***. ^19^F NMR (Toluene d_6_, 470 MHz, δ_ppm_) for Boc-D-(4F)Phe(1)-D-(4F)Phe(2)-COOMe (**2)**.

***Figure S15***. MALDI-TOF mass spectroscopy of Boc-D-(4F)Phe(1)-D-(4F)Phe(2)-COOMe (**2)**.

***Figure S16*.** ^1^H NMR (DMSO-*d*_6_, 400MHz, δ_ppm_) of Boc-L-Phe-COOH.

***Figure S17*.** ^1^H NMR (CDCl_3_, 400 MHz, δ_ppm_) Boc-L-Phe(1)-L-(4F)Phe(2)-COOMe (**3)**.

***Figure S18***. ^13^C NMR (CDCl_3_, 125 MHz, δ_ppm_) of Boc-L-Phe(1)-L-(4F)Phe(2)-COOMe (**3)**.

***Figure S19***. ^19^F NMR (Toluene d_6_, 470 MHz, δ_ppm_) for Boc-L-Phe(1)-L-(4F)Phe(2)-COOMe (**3)**.

***Figure S20***. ESI mass spectra of Boc-L-Phe(1)-L-(4F)Phe(2)-COOMe (**3)**.

***Figure S21*.** ^1^H NMR (CDCl_3_, 400MHz, δ_ppm_) of Boc-L-Phe(1) L-Phe(2)-COOMe (**4)**.

***Figure S22***. ^13^C NMR (CDCl_3_, 100MHz, δ_ppm_) of Boc-L-Phe(1) -L-Phe(2)-COOMe (**4)**.

***Figure S23***. MALDI-TOF mass spectroscopy of Boc-L-Phe(1)-L-Phe(2)-COOMe (**4)**.
